# Evaluation of Cell Models to Study Monocyte Functions in PMM2 Congenital Disorders of Glycosylation

**DOI:** 10.3389/fimmu.2022.869031

**Published:** 2022-05-06

**Authors:** Paola de Haas, Marien I. de Jonge, Hans J. P. M. Koenen, Ben Joosten, Mirian C. H. Janssen, Lonneke de Boer, Wiljan J. A. J. Hendriks, Dirk J. Lefeber, Alessandra Cambi

**Affiliations:** ^1^Department of Cell Biology, Radboud Institute for Molecular Life Sciences, Radboud University Medical Center, Nijmegen, Netherlands; ^2^Department of Laboratory Medicine, Laboratory of Medical Immunology, Radboud University Medical Center, Nijmegen, Netherlands; ^3^Department of Rehabilitation, Radboud Center for Mitochondrial Medicine, Radboud University Medical Center, Nijmegen, Netherlands; ^4^Department of Internal Medicine, Radboud Center for Mitochondrial Medicine, Radboud University Medical Center, Nijmegen, Netherlands; ^5^Department of Paediatrics, Radboudumc Amalia Children’s Hospital, Nijmegen, Netherlands; ^6^Department of Laboratory Medicine, Translational Metabolic Laboratory, Radboud Institute for Molecular Life Sciences, Radboud University Medical Center, Nijmegen, Netherlands; ^7^Department of Neurology, Donders Institute for Brain, Cognition and Behaviour, Radboud University Medical Center, Nijmegen, Netherlands

**Keywords:** glycosylation, monocytes, congenital disorders of glycosylation, immune cells, phosphomannomutase 2

## Abstract

Congenital disorders of glycosylation (CDG) are inherited metabolic diseases characterized by mutations in enzymes involved in different steps of protein glycosylation, leading to aberrant synthesis, attachment or processing of glycans. Recently, immunological dysfunctions in several CDG types have been increasingly documented. Despite these observations, detailed studies on immune cell dysfunction in PMM2-CDG and other CDG types are still scarce. Studying PMM2-CDG patient immune cells is challenging due to limited availability of patient material, which is a result of the low incidence of the disease and the often young age of the subjects. Dedicated immune cell models, mimicking PMM2-CDG, could circumvent many of these problems and facilitate research into the mechanisms of immune dysfunction. Here we provide initial observations about the immunophenotype and the phagocytic function of primary PMM2-CDG monocytes. Furthermore, we assessed the suitability of two different glycosylation-impaired human monocyte models: tunicamycin-treated THP-1 monocytes and PMM2 knockdown THP-1 monocytes induced by shRNAs. We found no significant differences in primary monocyte subpopulations of PMM2-CDG patients as compared to healthy individuals but we did observe anomalous surface glycosylation patterns in PMM2-CDG patient monocytes as determined using fluorescent lectin binding. We also looked at the capacity of monocytes to bind and internalize fungal particles and found a slightly increased uptake of *C. albicans* by PMM2-CDG monocytes as compared to healthy monocytes. Tunicamycin-treated THP-1 monocytes showed a highly decreased uptake of fungal particles, accompanied by a strong decrease in glycosylation levels and a high induction of ER stress. In contrast and despite a drastic reduction of the PMM2 enzyme activity, PMM2 knockdown THP-1 monocytes showed no changes in global surface glycosylation levels, levels of fungal particle uptake similar to control monocytes, and no ER stress induction. Collectively, these initial observations suggest that the absence of ER stress in PMM2 knockdown THP-1 cells make this model superior over tunicamycin-treated THP-1 cells and more comparable to primary PMM2-CDG monocytes. Further development and exploitation of CDG monocyte models will be essential for future in-depth studies to ultimately unravel the mechanisms of immune dysfunction in CDG.

## Introduction

Protein glycosylation, i.e. the covalent addition of glycan chains to specific amino acid residues in a peptide chain, is involved in a wide range of cellular processes as it affects folding, stability and function of many different proteins. Congenital disorders of glycosylation (CDG) is a group of more than 130 inherited metabolic diseases characterized by mutations in enzymes involved in different steps of protein glycosylation, resulting in problems in the synthesis, attachment or processing of glycans (1). The most common type is represented by patients with mutations in the cytosolic enzyme phoshomannomutase 2 (PMM2), which catalyzes one of the first reactions in the N-glycosylation pathway (2, 3).

Considering the importance of protein glycosylation, a very large variety of symptoms is associated with CDG. Neurological involvement is a common feature, which may include symptoms such as psychomotor retardation, hypotonia, microcephaly and stroke-like episodes. Other symptoms associated with CDG are hepatogastrointestinal disease, cardiac disease, renal disease, abnormal endocrine function, coagulopathy and skeletal abnormalities (4, 5).

Recently, it became increasingly clear that several CDG types are associated with immunological dysfunction, and SLC35C1-CDG, MAGT1-CDG and PGM3-CDG have even been classified as primary immunodeficiencies (6–8). Also PMM2-CDG is associated with recurrent and severe infections, which can have bacterial, viral or fungal causes (9, 10). Some PMM2-CDG patients have been reported to show altered immune cell populations, such as elevated white blood cell counts, increased amount of NK cells, decreased amount of T-cells, neutropenia and an expanded B cell compartment (11–14). Reduced immunoglobulin levels have also been found in multiple patients (15, 16). Moreover, it has been shown that stroke-like episodes occurring in 20-55% of PMM2-CDG patients are often triggered by viral infections (17). Complications of immune dysfunction can lead to reduced quality of life, hospitalization and in the worst case early mortality (9, 11).

Despite these observations, detailed studies addressing the mechanism of immune cell dysfunction in PMM2-CDG are scarce. Sporadic findings in PMM2-CDG patient immune cells have shown impaired neutrophil chemotaxis and increased NK cell responsiveness (11, 14). Studies in neutrophils and monocytes were suggestive of aberrant glycosylation of the membrane proteins CD16 and CD14 (18).

Studying PMM2-CDG patient immune cells is challenging due to limited sample availability, which is a result of the low incidence of the disease as well as the often young age of the subjects (19, 20). Furthermore, the high diversity of pathological mutations found in the *PMM2* gene and the variable disease severity introduce a bandwidth in the results and that complicates data interpretation. Dedicated immune cell models, that faithfully mimic PMM2-CDG, could circumvent many of these problems and facilitate research into the mechanism of immune dysfunction. A B cell model for PMM2-CDG has been established by EBV-transformation (21) and revealed an overall reduction of α2,6 sialylated glycans and a reduced binding to CD22 (21). Recently this model was also used to assess gene expression profiles showing altered expression of genes related to the stress response, glycosylation, cell junction and motility in PMM2-CDG patient derived cells (22).

In this study we will focus on innate immunity and compare different THP-1 monocyte models with PMM2-CDG monocytes. The THP-1 cell line was chosen as a monocyte model because of its versatility, since this cell line can be differentiated into macrophages, dendritic cell and osteoclasts, broadening the possible applications in CDG research (23–25). Different approaches were used to impair N-glycosylation in THP-1 cells, and effects on glycosylation level, Endoplasmic Reticulum (ER) stress, and pathogen uptake were determined in order to assess the value and limitations of these models for future research into the mechanisms of immune dysfunction in CDG.

## Materials and Methods

### Cell Culture

THP-1 cells (ATCC; no. TIB-202), derived from peripheral blood of an acute monocytic leukemia patient, were used as monocyte model. In addition, control PBMCs were isolated from apheresis products (Healthy 1, 2, 3) or full blood (Healthy 4-13) supplied by the blood bank Sanquin (Nijmegen) and CDG blood samples were collected from patients visiting the Radboudumc academic hospital (Nijmegen). The study was approved by the Institutional Review Board at Radboudumc (CMO-dossier number 2019-5654). Blood samples were taken on clinical indication and a part was used for this study after informed consent from either the patient or their legal representative, in accordance with the Declaration of Helsinki.

THP-1 cells and PBMCs were cultured in RPMI-1640 medium containing 25 mM HEPES (Gibco) and supplemented with 10% Fetal Bovine Serum (FBS) (Gibco) and 2 mM Ultraglutamine (Lonza). HEK293-FT cells (ATCC; no. PTA-5077), used for lentiviral production, were cultured in DMEM (Thermo Fisher Scientific) supplemented with 10% FBS (Gibco), 2 mM Glutamax (Gibco) and 1 mM Sodium Pyruvate (Gibco). All cell lines were cultured at 37°C in a humidified CO_2_ (5%) incubator. For the shRNA expressing cell lines 2 µg/mL of puromycin was added to the medium to maintain selection pressure.

### Tunicamycin Treatment

Cells were seeded at a density of 0.2 million/mL for control cells and 0.3 million/mL for tunicamycin (TM) treated cells, to correct for differences in proliferation rate between treatment groups. A concentration of 10 ng/mL TM (Abcam, stock 5 mg/mL in DMSO) or 0.0002% DMSO was used for the TM treatment and the control respectively, unless stated otherwise. After two days of culturing the cells were harvested, counted and reseeded at the starting density in fresh medium containing the relevant components. At day four of culturing the cells were harvested and used for experiments.

### shRNA Cell Lines

Lentiviral shRNA expression constructs specific for human *PMM2* transcripts were purchased as glycerol stocks (TRC clone ID: TRCN0000300512 and TRCN0000300514; Sigma-Aldrich). Bacterial cultures were grown from individual colonies and plasmids were isolated using a Plasmid Midiprep Kit (Qiagen). Lentiviral packaging was performed in HEK293-FT cells using the Virapower Lentiviral Expression system (Invitrogen). The day before transfection 3 million cells were seeded in a 10 cm petri dish in 10 mL of medium. The next day, 9 µg of ViraPower packaging mix and 3 µg of *PMM2* shRNA expression plasmid (or the Mission pLKO.1-puro Non-Target shRNA control; Sigma-Aldrich) were dissolved in 1.5 mL Optimem (Gibco). In a separate tube 36 µL Lipofectamine 2000 was diluted in 1.5 mL Optimem. Tubes were incubated for 5 m at room temperature whereafter plasmid and lipofectamine were mixed and incubated for another 20 m. Medium in the petri dish with HEK293-FT cells was replaced with 5 mL of fresh medium before the transfection mixture was gently added to the cells. The next day, medium was replaced with 10 mL fresh medium. Virus-containing supernatant was harvested 72 h after transfection and stored at -80°C until used.

For transduction purposes 1.2 million THP-1 cells were seeded in 2 mL of lentivirus-containing medium. Supernatant from both *PMM2* shRNA expression vectors TRCN0000300512 and TRCN0000300514 were used in combination (1:1 ratio). Cells were incubated overnight and the next day virus supernatant was removed and replaced by fresh medium. Two days later, 2 µg/mL of puromycin was added to select for transduced cells. Selective pressure was maintained during all subsequent culturing steps. Individual clonal lines were derived *via* limiting dilutions and screened for *PMM2* knockdown using qRT-PCR. Six to seven weeks after transduction the clonal derivatives were frozen and kept in liquid nitrogen for later use in experiments.

### PBMC Isolation

PBMCs were isolated by density gradient centrifugation. Blood in a EDTA collection tube was transferred to a 50 mL tube and the volume was adjusted to 35 mL with PBS. A layer of 14 mL lymphoprep (Stem Cell Technologies) was added under the diluted blood layer and the tube was centrifuged at 600 G for 30 m at room temperature with the brake off. The interphase layer containing the PBMCs was collected into another 50 mL tube and supplemented with cold PBS. The cells were centrifuged at 4°C for 15 m and this wash step was repeated two more times. The PBMCs were frozen and stored in liquid nitrogen until used in experiments.

### Lectin Staining

Some 50,000-200.000 cells were seeded in a round bottom 96 well plate which was kept on ice during the staining procedure. All centrifugation steps were done at 360 G for 2 m at 4°C. Cells were washed once in PBS and twice in carbo-free blocking solution (Vector Laboratories). Subsequently, the cells were stained with either Concanavalin A-biotin (50 µg/mL; Thermo Fisher Scientific), MAA-biotin (EY laboratories, 50 µg/mL), MALII-biotin (Vector laboratories, 30 µg/mL) or SNA-FITC (Vector laboratories, 30 µg/mL) in Carbo-free buffer supplemented with 1 mM CaCl_2_ and 1 mM MgCl_2_ for 30 m (FITC-labelled lectins) or 45 m (biotin-labelled lectins). After staining, the cells were washed three times in PBA (PBS with 1% FCS). Cells stained with biotin-labelled lectins were treated with streptavidin 5 µg/mL Alexa 488 (Thermo Fisher Scientific) for 10 m. Then the cells were washed three times in PBA and fluorescent intensity was measured immediately by flow cytometry. For the TM experiments the cells were stained just before measurement with 1 µg/mL propidium iodide (Miltenyi Biotec) as a viability dye. Alternatively the cells were washed in PBS and fixed in 2% PFA for 30 m. Fixative was washed out three times with PBS and the cells were kept in the fridge until measured by flow cytometry within one week of staining. Fixed cells from TM experiments were stained with Fixable Viability dye eFluor 780 (eBioscience, 2000x diluted) before lectin staining. PBMCs were stained with CD14 antibody (Thermo Fisher Scientific, clone 61D3, 5 µg/mL in PBA) for 20 m after lectin staining and the secondary antibody goat-anti-mouse H&L A647 (Thermo Fisher Scientific, 2 µg/mL) was added at the same time as streptavidin and incubated for 20 m. Samples from TM treatment experiments were measured on a MACSquant analyzer or a CyAn ADP analyzer while the shRNA and PBMC samples were measured on a BD FACSlyric flow cytometry system. Data were analysed using FlowJo10 software. Median fluorescence intensity (MFI) values were normalized to the average MFI (set as 1) of the control. Before normalization, the MFI values of the negative controls were subtracted. Percentage positive cells was quantified by gating the population with higher fluorescence intensity than the negative (unstained) control. The percentage obtained in the negative control samples was subtracted (varying between 0-17% per sample).

### qRT-PCR

For RNA isolation between 0.5 and 2 million cells were harvested by centrifugation and washed twice in PBS. The cell pellet was lysed in 350 µL RNA lysis solution and the suspension was put through an 18 gauge needle twelve times. The RNA lysate was either stored at -80°C until further processing or used for RNA isolation immediately. RNA was isolated with the Aurum Total RNA mini kit (Bio-Rad) and DNA contamination was removed with DNase I treatment. From the isolated RNA, 500 ng was used for cDNA synthesis with the iScript cDNA synthesis kit (Bio-Rad) in a total reaction volume of 20 µL. For each cDNA synthesis reaction a control without reverse transcriptase and a control without RNA template was taken along.

For quantitative RT-PCR, cDNA was diluted (80 times for *ATF4*, *HSPA5* and *DDIT3* and 25 times for *PMM2*) and 3 µL was mixed in a final volume of 10 µL containing 5 µL iQ SYBR Green Supermix (Bio-Rad) and 4 pmol of each primer. Primers used: *ACTB* Fw 5’-CGGGCCGTCTTCCCCTCCAT-3’ Rv 5’- TGGGCCTCGTCGCCCACATA-3’ (26), *GAPDH* Fw 5’-CCCGCTTCGCTCTCTGCTCC-3’ Rv 5’-CCTTCCCCATGGTGTCTGAGCG-3’ (26), *ATF4* Fw 5’-GTTCTCCAGCGACAAGGCTA-3’ Rv 5’- ATCCTGCTTGCTGTTGTTGG-3’ (27), *HSPA5* Fw 5’-TGTTCAACCAATTATCAGCAAACTC-3’ Rv 5’-TTCTGCTGTATCCTCTTCACCAGT-3’ (28), *DDIT3* Fw 5’-ACCTCCTGGAAATGAAGAGGAAG-3’ Rv 5’- CAGTCAGCCAAGCCAGAGAA-3’ (29) (Sigma-Aldrich). For *PMM2* knockdown measurement the Hs_PMM2_1_SG (QuantiText) primer assay was used in a 10 times dilution as recommended by the manufacturer. Samples were measured using a CFX96 Real-time System (Bio-Rad) set up to perform an initial 10 m at 95°C followed by 40 cycles of 15 s at 95°C and 50 s at 60°C. At the end a melt curve was made by increasing the temperature from 65°C-95°C at 1°C/s to confirm single product amplification.

### Internalization Assay

One million PBMCs or 0.1 million THP-1 cells were added to a V-bottom plate together with 2 million Alexa488 labelled zymosan A from *Saccharomyces cerevisiae* (Sigma-Aldrich) or with heat-killed *Candida albicans* in 20 µL of serum-free RPMI-1640 medium. After incubation at 37°C for the indicated timepoints, cells were gently transferred from the V-bottom plate to a poly-L-lysin coated coverslip and fixed in 4% PFA in 0.1 M sodium phosphate buffer (PB buffer) for 30 m. The fixation as well as all the steps of the staining procedure were performed at room temperature. Samples were washed twice with PBS and blocked in blocking buffer (PB buffer supplemented with 20 mM glycine) for 60 m. Thereafter, cell membranes were stained for 1 h using 5 µg/mL LFA-1 antibody (Clone TS2/4, hybridoma) or CD14 antibody (clone 61D3; Thermo Fisher Scientific) in blocking buffer. Samples were washed three times in PBS and stained with a secondary goat-anti-mouse-H&L Alexa647 antibody (Thermo Fisher Scientific) in blocking buffer for 60 m. Finally, samples were washed twice in PBS and once in PB buffer before being sealed in Mowiol and imaged on a Leica DMI6000 widefield microscope using a HCX PL APO 63X/1.40 oil Leica Microsystems objective.

### Immune Cell Populations

PBMC samples were thawed on the day of the assay. The staining and flow cytometry analyses was done as described previously using the ‘General panel’ from Aguirre-Gamboa et al. (30). In brief, PBMCs were stained in 25 µL of surface staining master mix containing CD14-ECD (clone RM052; Coulter) and CD16-FITC (clone 3G8; Coulter). Cells were washed twice and resuspended in 100 µL PBS containing 0.2% BSA. Then, cells were analysed using a 10-color Navios flow cytometer (Beckman Coulter) equipped with three solid-state lasers (488 nm, 638, and 405 nm). Data were analysed using Kaluza software version 1.3 (Beckman Coulter). CD14 positive cells were characterized as monocytes and subpopulations were identified by CD14 and CD16 expression.

### Enzyme Activity

For the PMM2 enzyme activity assay, 2.5 million cells were harvested and washed four times in PBS. Cell pellets were homogenized in a buffer containing 20 mM HEPES pH 7.1 (Sigma-Aldrich), 25 mM KCl, 1 mM dithiothreitol (Sigma-Aldrich), 10 µg/mL leupeptin (Roche), and 10 µg/mL antipain (FLUKA). Cells were disrupted by sonification and samples were centrifuged at 1550 G for 8 m and resulting supernatant was stored at -80°C until the enzyme activity assay was performed.

Supernatants were defrosted and centrifuged at 9000 G for 5 m. Total protein concentration was determined and PMM2 enzyme activity was measured spectrophometrically every 4.5 m at 30°C by the reduction of NADP to NADPH causing a change in A_340_. All of the reagents needed to convert mannose 1-phosphate to gluconate 6-phosphate and NADPH were added to the samples except for the enzyme measured in the assay, namely PMM2. The reaction solution consisted of 50 mM HEPES pH 7.1, 5 mM MgCl_2_, 2 µg/mL phosphoglucose isomerase (Roche), 1.7 µg/mL phosphomannose isomerase (Sigma-Aldrich), 10 µg/mL glucose 6-phosphate dehydrogenase (Roche), 1 µM mannose 1,6-bisphosphate, 0.25 mM NADP (Roche), and 0.34 mM mannose 1-phosphate (Sigma-Aldrich). As a negative control Mannose-1-phosphate was omitted from the sample and the extinction resulting from this was subtracted from the extinction measured in the sample in presence of the substrate. 1 U enzyme activity corresponds to the formation of 1 µmol of NADPH per m. The enzyme activity is normalized to the total amount of protein present in the sample (31).

### Statistics

Data are represented as mean ± standard deviation. Statistical analyses were performed using Graphpad Prism software 9.2.0. Primary monocyte and shRNA expressing THP-1 cell data were analyzed using an unpaired Student’s *t*-test. TM experiments were analyzed using a paired Student’s *t*-test, pairing data from TM treated cells with their respective untreated control. Results from the lectin binding assay in TM treated cells were analyzed using a ratio paired Student’s *t*-test because of varying lectin binding in untreated controls. Statistical significance of the different categories (binding and Internalization + binding) in the internalization assay was calculated separately by a One-way ANOVA and multiple comparisons.

## Results

### Patient Characteristics

The CDG patients included show an even distribution in gender (**Table 1**). Most patients were adults (>18) in the range between 25-50 years old but two younger patients were also included (ages 4 and 12 years old). The p.R141H and p.F119L mutation in PMM2, which are frequently reported in literature, were shared by some of the patients (32). However each patient in this cohort showed a unique *PMM2* genotype which included both heterozygous (4/7 patients) and homozygous mutations (2/7 patients). The overall disease severity as assessed by the patients’ clinician, varied from mild to very severe. Three out of seven patients had a history with recurrent or severe infections. For all the patients we performed a flow cytometry analysis of the monocyte subsets and whenever possible we determined the PMM2 residual enzymatic activity. Patient 1, 2 and 3 were chosen for a more detailed assessment of monocyte characteristics because they represent a broad range of age groups and the clinical symptoms were moderate to severe. In addition, patient 1 and 3 have a history of recurrent or severe infections while patient 2 has no noticeable immunological symptoms. This allows us to compare monocyte characteristics in patients with or without immune dysfunction.

**Table 1:** PMM2-CDG patient overview.

Patient	Age (in years)	Mutation	Gender	Recurrent or severe infections	Overall disease severity	PMM2 enzyme activity (in leukocytes; mU/mg total protein)
**PMM2-CDG 1**	4	p.P113L, p.R141H	F	Yes	Severe	0.04
**PMM2-CDG 2**	26	p.Y64S, p.R21G	M	No	Moderate	Unknown
**PMM2-CDG 3**	41	p.R141H, p.F119L	M	Yes	Severe	0.0
**PMM2-CDG 4**	12	p.F119L homozygous	F	No	Very severe	0.14
**PMM2-CDG 5**	28	Unknown *	M	Yes	Moderate	Unknown *
**PMM2-CDG 6**	34	p.R141H, p.G186R	F	No	Moderate	0.0
**PMM2-CDG 7**	47	p.K51R homozygous	F	No	Mild	0.09
**Healthy controls** **N = 56**	–	–	–	–	–	Average: 2.5 Range: 0.47-5.31 (see **Figure 1E**)

^*^Diagnostics performed in another hospital, data not accessible to us.

### PMM2-CDG Monocyte Populations and Glycosylation Levels

**Figure 1:**
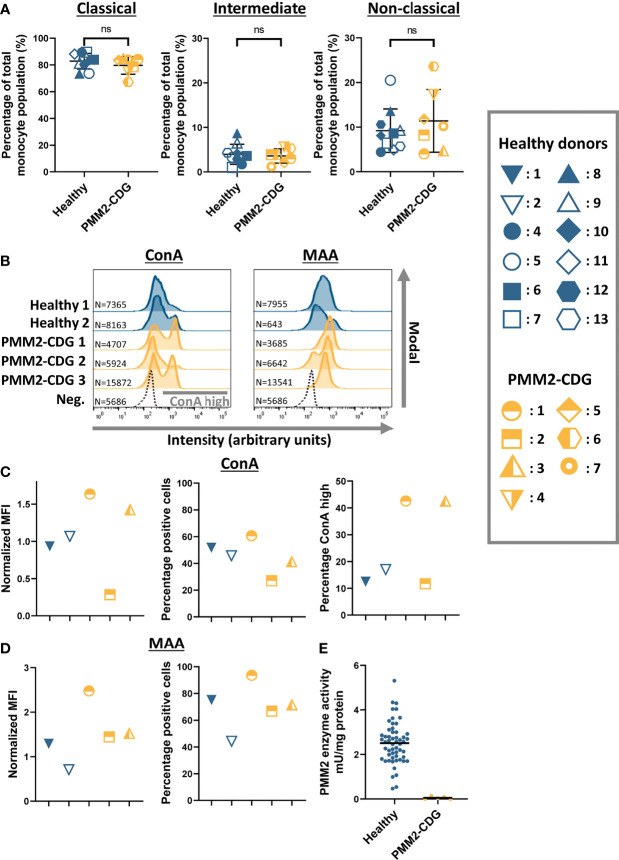
Characterization of monocytes from PMM2-CDG patients **(A)** The relative abundance of monocyte subpopulations divided into classical (CD14^++^CD16^-^), intermediate (CD14^+^CD16^+^) and non-classical (CD14^++^CD16^+^) monocytes in healthy control (n = 10) and PMM2-CDG patient PBMCs (n = 7). **(B)** Histogram plots showing the intensity of fluorescently labelled lectins (ConA and MAA) bound to monocytes (CD14+) on a logarithmic scale. The modal is used to represent the data which scales the plot as the proportion of the maximum count. Absolute cell numbers (N) are indicated. For simplicity, here we indicate only the unlabelled negative control (Neg.) related to sample Healthy 2 as representative plot. **(C, D)** Quantification of the histogram plots showing the median fluorescent intensity (MFI) normalized to the average MFI (set as 1) of the healthy controls (C ConA; D MAA). Before normalization, the MFI values of the negative controls were subtracted. ConA binding resulted in some conditions in two defined subpopulations (see grey bar in B), the percentage of ConA high from the total cell population is also plotted. **(E)** PMM2 enzyme activity in leukocytes isolated from healthy donors (n = 56) and PMM2-CDG patients (n = 5). Results are summarized in **Table 1**. Mean ± S.D. Significance was calculated by unpaired Student’s t-test **(A)**. ns, non-significant.

For this study peripheral blood mononuclear cells (PBMCs), were isolated from blood of PMM2-CDG patients and healthy donors. To assess the abundance of different monocyte populations, monocytes were phenotypically divided in classical (CD14^++^CD16^-^), intermediate (CD14^+^CD16^+^) and non-classical (CD14^++^CD16^+^) monocytes. Classical (CD14^++^CD16^-^) monocytes are most abundant in blood and are described to be highly phagocytic while non-classical (CD14^++^CD16^+^) monocytes are specialized in complement and FcR-mediated phagocytosis as well as anti-viral responses. Intermediate (CD14^+^CD16^+^) monocytes form the transition state between classical and non-classical cells and function in antigen presentation (33). The abundance of subpopulations is expressed as a percentage of the total monocyte population (**Figure 1A**). As expected, most monocytes (around 80%) were of the classical (CD14^++^CD16^-^) subgroup, while the non-classical (CD14^++^CD16^+^) and intermediate (CD14^+^CD16^+^) monocytes represented around 10% and 5% of the circulating monocytes respectively. There were no significant differences found in the abundance of monocyte populations between PMM2-CDG patient and healthy donors.

Next, we determined the global glycosylation level at the cell membrane of monocytes from healthy donors and PMM2-CDG patients. We performed a lectin surface binding assay using two fluorescently labeled lectins, namely Concanavalin A (ConA), which binds to α-D-mannose and α-D-glucose structures, and *Maackia amurensis agglutinin* (MAA), which binds to α2-3-sialylated glycans, after optimizing their concentration range using THP-1 monocytes (**Supplementary Figure 1**). PBMCs were double stained for the CD14 marker and with either ConA or MAA and analyzed by flow cytometry (**Figures 1B**–**D**; **Supplementary Figure 2**). Histogram plots of the fluorescence intensity show binding of ConA and MAA to monocytes of all the healthy donors and PMM2-CDG patients (**Figure 1B**). However not all monocytes in one sample bind to these lectins, as can be observed by the percentage positive cells (**Figures 1C, D**). Interestingly, in the ConA histogram plots two distinct monocyte populations become visible in patient 1 and 3 material, more than 40% show high ConA binding. In the patient 2 sample, however, only a single monocyte group is detected, which has a lower overall ConA binding compared to healthy donor material (**Figure 1C**). All the PMM2-CDG patient monocytes show a trend towards a higher MAA binding compared to healthy donor controls, which is most prominent in patient 1-derived cells (**Figure 1D**).

Since only minor differences in glycosylation levels were observed between healthy control monocytes and PMM2-CDG monocytes, we were interested in the PMM2 enzyme activity in immune cells isolated from PMM2-CDG patients. PMM2 enzyme activity was measured in leukocytes of five patients included in this study (**Table 1** and **Figure 1E**). The PMM2 enzyme activity in healthy control leukocytes varied between 0.47-5.31 mU/mg total protein and was on average 2.5 mU/mg. In PMM2-CDG patients, however, the enzyme activity was severely reduced: in two patients the activity was undetectable, while in the other patients there was between 1.6 and 5.6% residual activity, compared to the average in healthy controls.

### Healthy and PMM2-CDG Monocytes Are Equally Able to Bind and Phagocytose *C. albicans*

**Figure 2:**
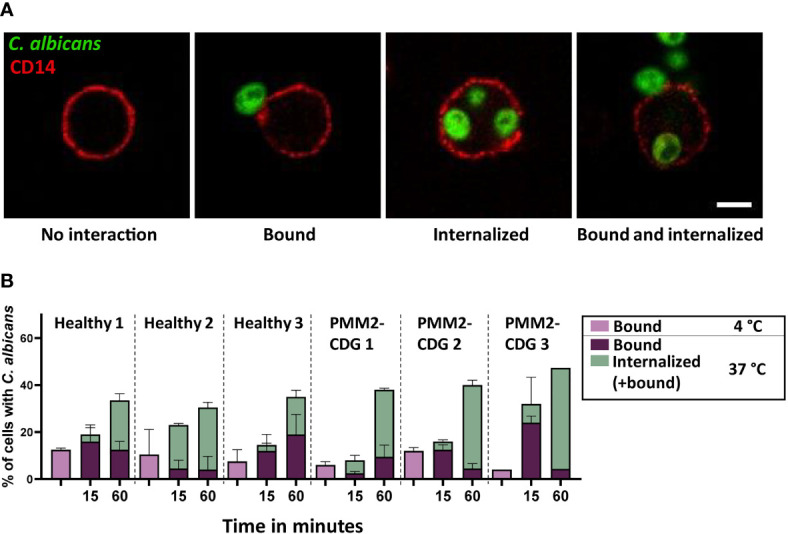
Binding and phagocytosis by PBMCs of healthy donors and PMM2-CDG patients. **(A)** Confocal microscopy images of monocyte interactions with *C. albicans*. The cell membrane is labelled by CD14 staining. Scale bar = 5 µm **(B)** Binding and uptake of *C. albicans* by healthy and PMM2-CDG patient monocytes after 15 or 60 m coincubation at 37°C. Samples incubated for 60 m at 4°C were included as a negative control. The experiment was performed in duplicate, except for the PMM2-CDG 3, 60 m at 37°C condition which includes one measurement. Mean ± S.D.

An important function of monocytes is patrolling the tissue for recognition and uptake of invading pathogens. We used fluorescently labeled heat killed *C. albicans* to compare the phagocytic capacity of healthy and PMM2-CDG monocytes. We incubated PBMCs with *C. albicans* for different periods of time (15 m and 60 m) and at either 4°C (to allow only binding) or 37°C (to allow binding and internalization). The samples were then washed and fixed, and the PBMCs were stained for membrane protein CD14, which reveals the cell membrane and functions as a monocyte marker. The interaction of the monocytes with the pathogen was visualized with fluorescent microscopy and the cells were manually counted and scored as either ‘bound’ or ‘internalized’, representing cells with *C. albicans* in close contact with the cell membrane or inside the cytosol respectively (**Figure 2A**).

After 15 m of incubation with *C. albicans* only a small percentage of monocytes had internalized the pathogen, varying between 0-20%, and there were no noticeable differences between healthy donor and PMM2-CDG monocytes (**Figure 2B**). In contrast, after 60 m of incubation a higher percentage of PMM2-CDG patient monocytes had taken up *C. albicans* compared to the healthy donor controls, even though pathogen binding was comparable. This increase in uptake was not dramatic but observed in all three patient cells and most striking in patient 3.

### Tunicamycin Treatment as a Model for PMM2-CDG

**Figure 3:**
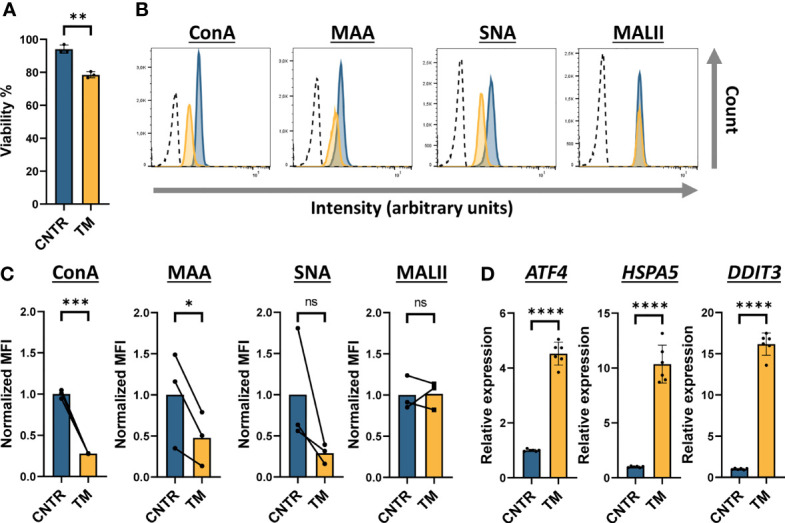
Tunicamycin-treated THP-1 monocytes as a model to mimic PMM2-CDG THP-1 cells were treated with 10 ng/mL tunicamycin for 4 days **(A)** Viability of tunicamycin (TM) treated THP-1 cells assessed by flow cytometry after staining with propidium iodide (PI) (n = 3). **(B)** Histogram plots showing the intensity of fluorescently labelled lectins (ConA, MAA, SNA and MALII) bound to THP-1 cells on a logarithmic scale. For simplicity, here we indicate only the unlabelled negative control (dotted line) related to sample CNTR as representative plot. **(C)** Quantification of the histogram plots showing the median fluorescent intensity (MFI) normalized to the average MFI (set as 1) of the control (CNTR) (n = 3). Before normalization, the MFI values of the negative controls were subtracted. **(D)** Assessment of the ER stress response by measuring the RNA expression level of three ER stress components *ATF4, HSPA5* and *DDIT3* by qRT-PCR. The data was normalized to *ACTB* and *GAPDH* expression. Significance was calculated by a paired student’s t-test **(A, D)** or a ratio paired student’s t-test **(C)**. Mean ± S.D., ns, non-significant, * ≤ p-value 0.05, **≤0.01, ***≤0.001, ****≤0.0001.

PMM2-CDG is a rare disease and fresh blood from these patients is not always available. To mimic the disease phenotype in a monocyte cell model we inhibited glycosylation *via* tunicamycin (TM) treatment in the human THP-1 monocytic cell line. TM blocks the first step in the formation of the lipid-linked oligosaccharide (LLO) needed for N-glycosylation by inhibiting GlcNAc phosphotransferase. For this reason TM is broadly used to study the effect of hypoglycosylation (34–36). Literature suggests that complete absence of PMM2 enzyme activity is not compatible with life and therefore some residual activity remains in all PMM2-CDG patients (32, 37). To simulate this aspect of PMM2-CDG we aimed to find the minimal dose of TM treatment of THP-1 cells that interfered with glycosylation but had a limited impact on cell viability. The optimal TM treatment of THP-1 cells was established to be four days of treatment with 10 ng/mL TM (**Supplementary Figure 3**). This treatment was used for the rest of this study and the effect of TM treatment on the viability, surface glycosylation level and ER stress level was assessed (**Figure 3**).

Although TM treatment increased the number of dead cells, the viability of the cells was still very high (**Figure 3A**).The membrane glycosylation was significantly altered by TM treatment as a 70% reduction in ConA and 50% reduction of MAA binding to the cell surface was observed (**Figures 3B, C**). In addition, binding of the cell to *Sambucus nigra* (SNA) lectin, which preferentially binds to α2-6-sialylated glycans, appeared to be lower, albeit not statistically significant, after TM treatment. To determine whether TM specifically affected N-glycosylation and not O-glycosylation, we determined THP-1 binding to the fluorescent *Maackia amurensis* lectin II (MALII), which binds to α2-3-sialylated O-glycans (38). No significant change in MALII binding between TM treated and control cells was observed, strongly suggesting that the TM treatment only affected N-glycosylation in THP-1 monocytes.

Since TM is a known ER stress inducer and glycosylation is important in protein folding and quality control, we determined to what extent TM treatment induced ER stress in THP-1 monocytes. To this aim, we measured RNA expression of three ER stress markers, *ATF4*, *HSPA5* and *DDIT3* by RT-qPCR (**Figure 3D**). RNA expression of all these markers was significantly increased after TM treatment compared to the control, the effect was strongest on *DDIT3* expression where a 16-fold increase of RNA expression was observed in TM treated cells. To determine whether the ER stress measured in these TM treated THP-1 cells affected cell viability on a long term, we incubated THP-1 cells with different concentrations of TM for up to three weeks and found that more than 70% of the cells were still viable and maintained reduced N-glycosylation levels (**Supplementary Figure 3**). Based on these results, we established the conditions under which a minimum amount of TM was found to preserve viability, induce a tolerable ER stress level while significantly decreasing N-glycosylation in THP-1 monocytes.

### shRNA-Induced PMM2 Knockdown as a Model of PMM2-CDG

**Figure 4:**
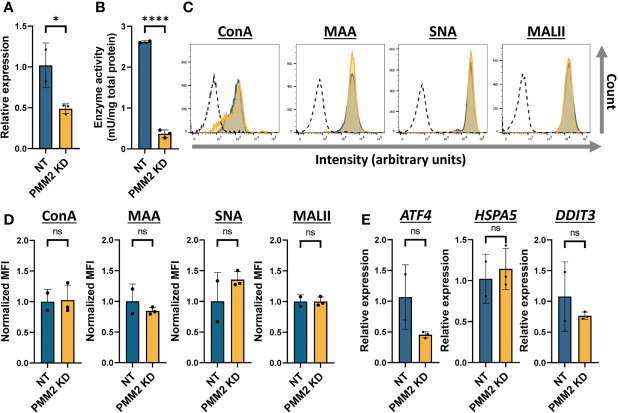
shRNA-induced PMM2 knockdown as a model of PMM2-CDG **(A)** Quantification of the *PMM2* RNA expression in THP-1 cells expressing non-targeting (NT, n = 2) or PMM2-targeting (PMM2 KD, n = 3) shRNAs. The mRNA expression level of *PMM2* was measured by qRT-PCR and the data was normalized to *ACTB* and *GAPDH* expression. **(B)** The PMM2 enzyme activity in NT (n = 2) and PMM2 knockdown THP-1 cells (n = 3). **(C)** Histogram plots showing the intensity of fluorescently labelled lectins (ConA, MAA, SNA and MALII) bound to THP-1 cells on a logarithmic scale. For simplicity, here we indicate only the unlabelled negative control (dotted line) related to sample NT as representative plot. **(D)** Quantification of the histogram plots showing the median fluorescent intensity (MFI) normalized to the average MFI (set as 1) of the NT. Before normalization, the MFI values of the negative controls were subtracted. **(E)** Assessment of the ER stress response by measuring the RNA expression level of three ER stress components *ATF4, HSPA5* and *DDIT3* by qRT-PCR. The data was normalized to *ACTB* and *GAPDH* expression. Significance was calculated by an unpaired student’s t-test. Mean ± S.D., ns, non-significant, * ≤ p-value 0.05, ****≤0.0001.

As an alternative to TM treatment, we knocked down *PMM2* expression in THP-1 cells using shRNAs *via* stable lentiviral transduction. First, we determined the reduction in *PMM2* mRNA levels in these knockdown cells and found about 50% decrease (**Figure 4A**). To determine whether the shRNA-induced reduction in PMM2 mRNA indeed affected PMM2 activity, we prepared cell lysates and performed a PMM2 enzyme activity assay. We found a highly significant decrease in PMM2 enzymatic activity that went from 2.62 mU/mg in the non-targeting (NT) control cells to 0.37 mU/mg in PMM2 knockdown cells. This corresponds to about 14% residual enzymatic activity in PMM2 knockdown cells, which is in line with the residual enzymatic activity previously reported for CDG patient cells (**Figure 4B**) (31, 39). We next determined the overall glycosylation level by fluorescent lectin binding assays but, surprisingly, none of these lectins showed an altered binding in PMM2 knockdown cells compared to the NT cells (**Figures 4C, D**). We also measured ER stress in these PMM2 knockdown cells by analyzing the RNA expression levels of the ER stress markers, *ATF4*, *HSPA5* and *DDIT3*, but these were not significantly impacted by the PMM2 knockdown (**Figure 4E**).

Collectively, these data suggest that PMM2 knockdown THP-1 cells may better resemble the primary monocytes of CDG patients than TM-treated monocytes.

### Phagocytosis in PMM2-CDG Monocyte Models

**Figure 5:**
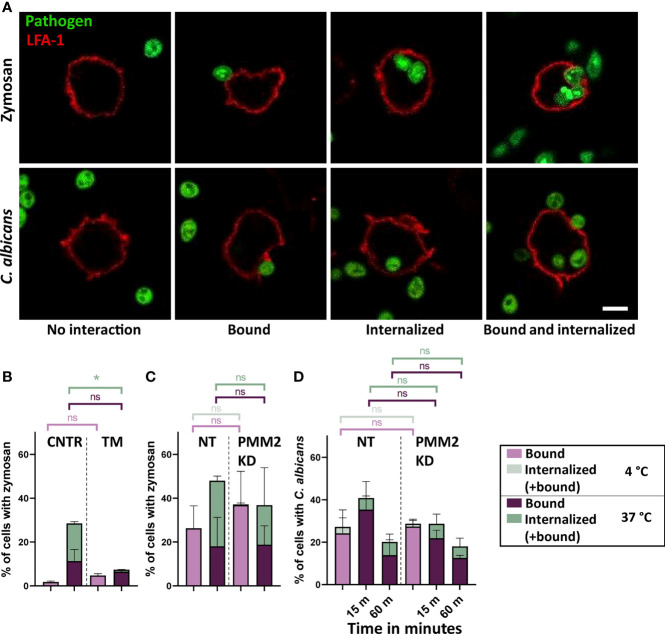
Phagocytosis in glycosylation impaired THP-1 cells **(A)** Confocal microscopy images of THP-1 cell interactions with zymosan and *C. albicans*. The cell membrane is labelled by LFA-1 staining. Scale bar = 5 µm. **(B)** Binding and uptake of zymosan particles by control (CNTR, n = 2) and tunicamycin (TM, n = 2) treated THP-1 cells after 90 m of coincubation at 4°C and at 37°C. Cells were treated with 10 ng/mL tunicamycin for 4 days **(C)** Binding and uptake of zymosan particles by cells expressing non-targeting (NT, n = 2) or PMM2-targeting (PMM2 KD, n = 2) shRNAs after 90 m of coincubation at 4°C and at 37°C. **(D)** Binding and uptake of *C*. *albicans* by NT (n = 2) and PMM2 KD (n = 3) THP-1 cells after 15 or 60 m coincubation at 37°C. Negative control samples were incubated for 60 m at 4°C. Significance was calculated by One-way ANOVA and multiple comparisons. Mean ± S.D., ns = non-significant, * ≤ p-value 0.05.

To test the phagocytic function of glycosylation impaired THP-1 cells, we performed an internalization assay using fluorescent zymosan, cell wall particles derived from *S. cerevisiae*, and heat killed *C. albicans* as model pathogens. THP-1 cells were incubated with these fungal particles for different periods of time (15 m, 60 m or 90 m) at 4°C (to allow binding only) or 37°C (to allow binding and internalization). After fixation the cells were stained with an antibody against the leukocyte specific integrin LFA-1 to visualize the membrane by fluorescent microscopy. The binding and internalization events were manually counted as described for the internalization assay with PMM2-CDG patient monocytes (**Figure 5A**).

The binding and uptake of fluorescent zymosan particles by TM treated and PMM2 knockdown THP-1 monocytes were surprisingly quite different (**Figures 5B, C**). TM treated monocytes showed a very strong decrease in zymosan uptake compared to the untreated cells, even after 90 m of incubation at 37°C (**Figure 5B**), whereas PMM2 knockdown THP-1 monocytes showed a similar binding and uptake as the controls, i.e. cells treated with NT shRNAs (**Figure 5C**).

For the PMM2 knockdown THP-1 monocytes, phagocytosis of *C. albicans* was measured, using the protocol for the PMM2-CDG patient PBMCs (**Figure 5D**). With respect to zymosan, similar binding levels but clearly less uptake of *C. albicans* were observed. No differences were observed between NT and PMM2 knockdown THP-1 monocytes. When compared to the phagocytosis of *C. albicans* by the monocytes of PMM2-CDG patients (**Figure 2**), the PMM2 knockdown THP-1 monocytes hardly showed any uptake.

In summary, the glycosylation impaired THP-1 monocytes showed a very heterogeneous ability to internalize zymosan particles or *C. albicans* and neither the TM-treated nor the PMM2 knockdown monocytes were found to recapitulate the behavior of PMM2-CDG patient monocytes.

## Discussion

In this study we analyzed monocyte phenotype and function in PMM2-CDG patients and assessed the value and suitability of two potential human PMM2-CDG monocyte models: TM-treated THP-1 monocytes and PMM2 knockdown THP-1 monocytes.

The distribution over monocytic cell types in PBMC samples from PMM2-CDG patients grossly matched that in control samples. Glycosylation patterns, however, were distinct and surprisingly also differed between patient samples. Monocytes from two out of three patients (patient 1 and 3) showed increased binding of ConA in a subset of cells while monocytes from one patient (patient 2) showed an overall decreased ConA binding compared to healthy donors. Interestingly, patients 1 and 3 have a severe clinical phenotype with immune involvement including recurrent or severe infections which most often were of bacterial origin. In patient 1 infections included a single occurrence of orbital cellulitis and endocarditis. In contrast, the symptoms of patient 2 were considered moderate and this patient did not experience recurrent or severe infections, suggesting a possible correlation between changes in glycosylation level and these clinical features. Moreover monocytes from all patients showed a small increase in binding to MAA.

Based on disease pathology, one might expect a decrease in lectin binding in PMM2-CDG patient cells, due to hypoglycosylation. In fact, hypoglycosylation of serum transferrin is used to diagnose patients with CDG and reduced lectin binding has been observed in PMM2-CDG patient derived platelets, induced pluripotent stem cells and EBV transformed B cells (22, 40–42). Eventhough it was not addressed in this study, the increased lectin binding in PMM2-CDG patient monocytes that we observed could be explained by subtle differences in activation status that alter glycoprotein expression and therefore glycocalyx composition. This aspect should be further investigated as it has been previously reported in human T cells (43). The presence of two distinct monocyte subpopulations which differ in ConA binding in two PMM2-CDG patient samples suggest that monocytes can be differently affected by the PMM2 mutation. The fact that we found increased as well as decreased binding of ConA in PMM2-CDG patient monocytes compared to healthy controls suggests that multiple mechanism, such as hypoglycosylation and possibly altered glycoprotein expression, might be at play depending on disease severity and symptoms. Such a compensatory mechanism has been described in HL-60 leukemia cells which show increased expression of the glutamine transporter, the glycoprotein SLC1A5, after induction of hypoglycosylation by TM (44). Finally, another explanation is provided by recent evidence suggesting that CDG-I defects, including PMM2-CDG, do not merely result in hypoglycosylation but also in glycan structural changes, including an elevated expression of short high-mannose N-glycans (45, 46). This could possibly contribute to the high lectin signal detected in some of the PMM2-CDG PBMCs. Performing the lectin binding assay with a larger group of PMM2-CDG patients could help in determining whether the observed effect is a general feature of PMM2-CDG monocytes. Another possibility is that detectable changes in glycosylation levels occur when the cells are under a certain pressure, such as induced proliferation, development or differentiation.

In TM treated THP-1 monocytes, however, a clear decrease in binding of ConA and MAA was observed as well as a trend towards reduced binding of SNA. Binding of MALII was not affected confirming that N-glycosylation but not O-glycosylation is specifically inhibited by TM treatment (38). A reduction in lectin binding on TM treated cells was therefore expected. In contrast, upon shRNA-mediated knockdown of PMM2 activity in THP-1 cells, we did not observe aberrant lectin binding of ConA, MAA or SNA. Similar to TM treatment, PMM2 knockdown impairs the initial steps of N-glycosylation and prevents formation of the LLO. This suggests that TM treatment more severely impacts N-glycosylation than PMM2 knockdown in THP-1 monocytes.

As a consequence of being a glycosylation inhibitor, TM is also known as a ER stress inducer since hypoglycosylation causes accumulation of misfolded proteins (47). The ER stress in TM-treated monocytes was indeed significantly increased compared to the control cells. Although ER stress is also expected in PMM2-CDG patient cells to some extent, it has been shown in fibroblasts that TM treatment impacts ER stress more severely than mutations in PMM2 (48). This would explain the absence of ER stress in PMM2 knockdown monocytes. In addition, it has been reported that TM suppresses TLR-induced inflammation in a mechanism independent from N-glycosylation or ER stress, suggesting that TM treatment impacts other cell processes besides N-glycosylation (49). Altogether, although TM is widely used as specific N-glycosylation inhibitor, its indirect but global effects on cell functioning urge for caution in interpreting results.

Interestingly, when looking at the phagocytic capacity of PMM2-CDG monocytes, we found a modest but clear increase in the uptake of *C. albicans* in PMM2-CDG patient monocytes. Monocytes recognize *C. albicans via* mannosylated glycans, β-glucan and mannan structures on the fungal membrane using multiple receptors from the Toll like receptor (TLR) and C-type lectin (CTL) family (50). All of these receptors contain putative N-glycosylation sites and therefore their glycosylation could be affected in PMM2-CDG.

For a few of these receptors it has been shown that hypoglycosylation impacts function, such as TLR-2 and TLR-4 which need their glycans for secretion and LPS receptor functioning, respectively (51, 52). In contrast to our finding, glycosylation of the *C. albicans* binding receptor, dectin-1, has been shown to be important for proper cell membrane expression (53, 54), suggesting possibly reduced levels of dectin-1 expression on PMM2-CDG monocytes which could lead to lower pathogen binding ability. However, dectin-2 and mincle work in collaboration with dectin-1 to control *C. albicans* infection (55–57). Both dectin-2 and mincle contain multiple putative N-glycosylation sites but the effect of hypoglycosylation on these receptors is currently unknown. Future studies will have to determine whether glycosylation of these pathogen-recognition receptors is changed in PMM2-CDG monocytes and how the combined effect of altered receptor glycosylation could impact pathogen binding and uptake. Interestingly, it has been shown that a thick glycocalyx could prevent particles from binding to the phagocytic receptors (58). It remains to be determined whether a thinner glycocalyx is present on PMM2-CDG monocytes that could provide easy access of pathogen, thus explaining our initial results.

THP-1 treatment with TM significantly reduced phagocytosis of zymosan, which can be recognized by different pathogen recognition receptors, including TLR-2 and Dectin-1 (59, 60). Two isoforms of Dectin-1 exist, isoform A and isoform B, that can bind fungal β-Glucan and which differ in the presence or absence of a stalk region containing a N-glycosylation site, respectively. Isoform A is better capable to bind zymosan and the N-glycan is needed for its membrane expression, possible explaining the results obtained in our study (54). In contrast, phagocytosis in PMM2 knockdown THP-1 monocytes was not impaired compared to NT control cells. This might be explained by the more severe impact of TM on membrane N-glycosylation and/or ER stress as compared to PMM2 knockdown, likely resulting in differences in pathogen receptor glycosylation between both models. Supporting this hypothesis, it has been suggested that ER stress in monocytes isolated from diabetes patients attenuated phagocytic capacity and TM treatment of healthy monocytes impaired the response to TLR ligands (61).

Even though the PMM2 enzyme activity in PMM2 knockdown THP-1 monocytes was in line with the reported residual activity in PMM2-CDG patients (31, 39), the increased uptake of *C. albicans* found in PMM2-CDG monocytes was not observed in the PMM2 knockdown CDG model. This could be explained by the difference in effect caused by strongly reduced expression versus mutations which result in loss of function as found in the PMM2-CDG patients. Despite the intrinsic challenges of gene editing in immune cell lines it will certainly be possible in the future to create THP-1 or other cell lines that mimic exactly the mutations found in PMM2-CDG patients (62, 63). Another possible explanation is the intrinsic differences between primary monocytes and THP-1 cells (64). Despite these limitations, we recommend the shRNA PMM2 knockdown model over the TM treatment model, since this can be further optimized to better mimic the most severe PMM2 phenotypes, more closely resembles the disease mechanism and circumvents the problems of unwanted side effects.

Collectively, our study offers an initial characterization of primary monocytes isolated from PBMCs of a group of PMM2-CDG patients as well as a first comparison between these primary PMM2-CDG monocytes and glycosylation-impaired THP-1 monocytes models. Even though not all phenotypes and functions of CDG patient monocytes could be recapitulated using THP-1 cells, such *in vitro* preclinical models are still valuable to study the mechanisms underlying immune cell dysfunction in CDG. Future efforts should be made to better characterize the *PMM2* knockdown THP-1 monocytes and possibly to create a genetic model that fully mimics the PMM2 mutations found in patients and use all these immune cell models to investigate several aspects of immunological dysfunctions in CDG patients.

## Data Availability Statement

The original contributions presented in the study are included in the article/**Supplementary Material**. Further inquiries can be directed to the corresponding author.

## Ethics Statement

CDG blood samples were collected from patients visiting the Radboudumc academic hospital (Nijmegen). The study was approved by the Institutional Review Board at Radboudumc (CMO-dossier number 2019-5654). Blood samples were taken on clinical indication and a part was used for this study after informed consent from either the patient or their legal representative, in accordance with the Declaration of Helsinki. Written informed consent to participate in this study was provided by the participants’ legal guardian/next of kin.

## Author Contributions

PdH designed and executed the experiments, analyzed the data and wrote the manuscript. MdJ and HK performed the PMM2-CDG PBMC phenotyping and analyzed the data. BJ provided technical assistance in the experiments. MJ and LdB coordinated the contact with the CDG patients and the collection of the blood samples. WH provided expertise on the shRNA experiments and data analysis. DL provided expertise on the CDG patients and supervised the project. AC designed, coordinated and supervised the project. All authors contributed to data analysis and interpretation and to the writing of the manuscript.

## Funding

Research described in this study was financially supported by a Radboudumc Intramural grant (to AC) and by Stofwisselkracht grant R0005003 (to AC and PH).

## Conflict of Interest

The authors declare that the research was conducted in the absence of any commercial or financial relationships that could be construed as a potential conflict of interest.

## Publisher’s Note

All claims expressed in this article are solely those of the authors and do not necessarily represent those of their affiliated organizations, or those of the publisher, the editors and the reviewers. Any product that may be evaluated in this article, or claim that may be made by its manufacturer, is not guaranteed or endorsed by the publisher.
