# Supplementary material for: Evaluation of Cell Models to Study Monocyte Functions in PMM2 Congenital Disorders of Glycosylation
Source: Front Immunol. 2022 May 6;13:869031. doi: 10.3389/fimmu.2022.869031 (PMC9121068; doi:10.3389/fimmu.2022.869031)
Supplement: Supplementary file 1 [file DataSheet_1.pdf]

Supplementary Material

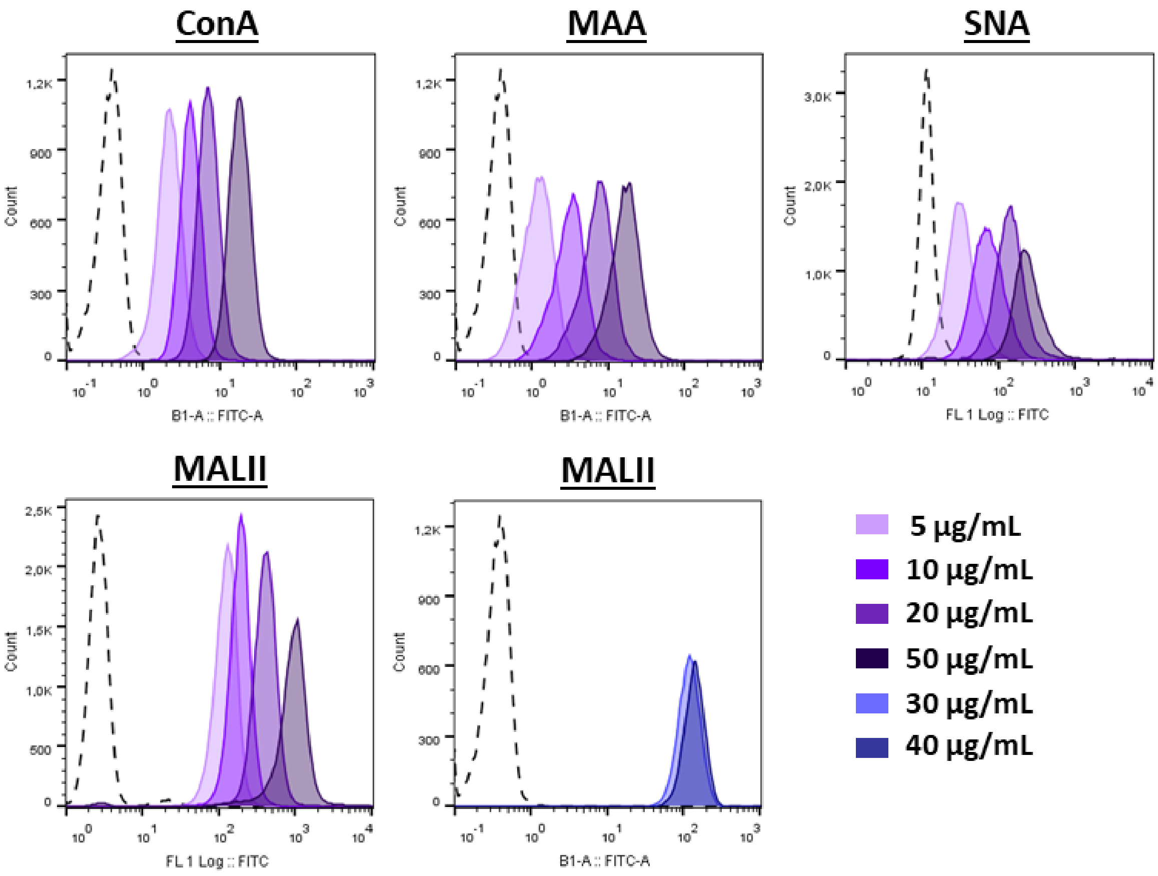

Supplementary Figure 1: Determining the optimal lectin concentration for staining. Different concentrations of lectins were used to stain untreated THP-1 cells. Histogram plots show the intensity of fluorescently labelled lectins (ConA, MAA, SNA and MALII) on a logarithmic scale, as measured by flow cytometry. Based on these results we decided to use the following lectin concentration for further stainings: ConA (50 µg/mL), MAA (50 µg/mL, SNA (30 µg/mL) and MALII 30 µg/mL.

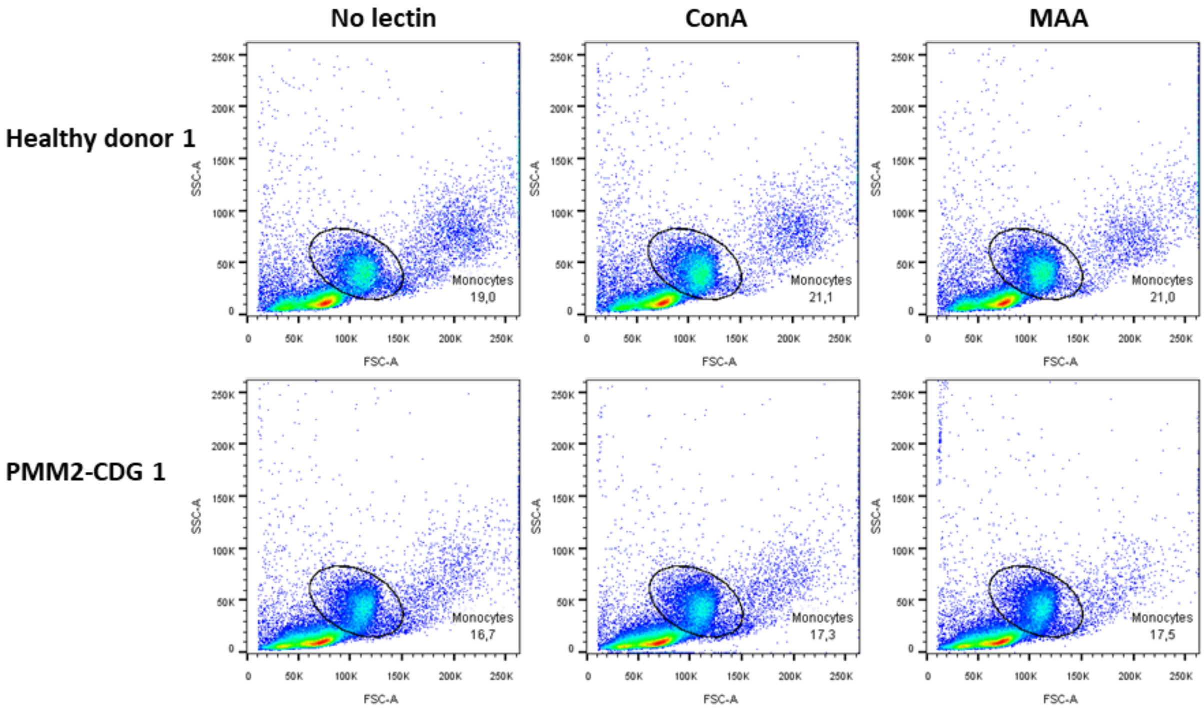

Supplementary Figure 2: Size and granularity of healthy control and PMM2-CDG PBMCs. Dot plots showing the Side scatter (SSC-A) and the Forward scatter (FSC-A) of healthy and PMM2-CDG PBMCs on a linear scale before and after lectin staining, as measured by flow cytometry. After gating the monocytes, as depicted in this plot, CD14+ cells were selected and used to measure lectin binding (results in figure 1 B, C and D). No signs of increased cell death after lectin incubation are visible.

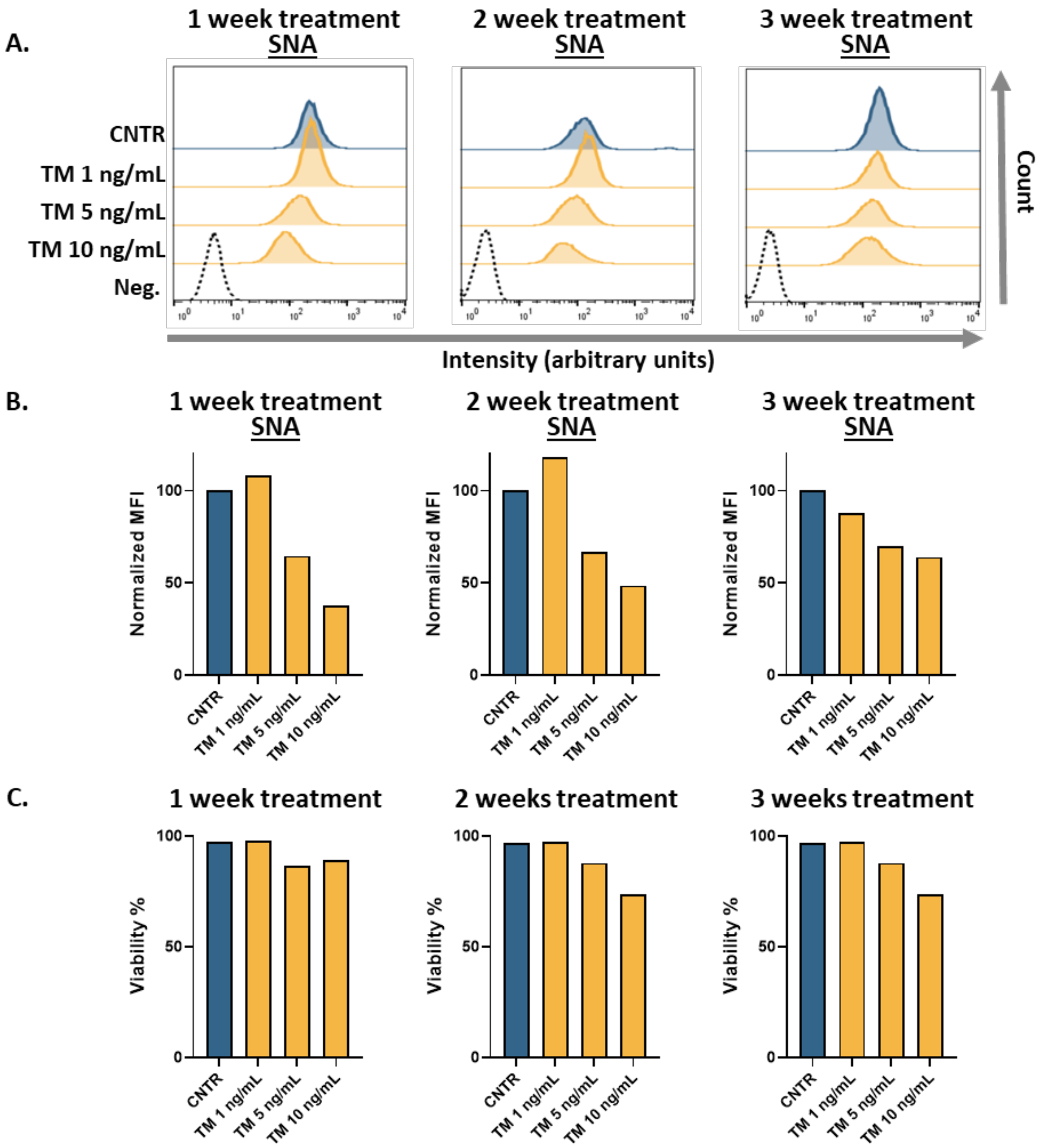

Supplementary Figure 3. Long term tunicamycin treatment in THP-1 cells. **(A)** Histogram plots showing the intensity of the fluorescently labelled lectin, SNA, bound to 1, 5 and 10 ng/mL tunicamycin (TM) treated or untreated control (CNTR) THP-1 cells on a logarithmic scale. SNA binding was measured after one, two and three weeks of treatment (n=1). For simplicity, here we indicate only the unlabelled negative control (dotted line) related to sample CNTR as representative plot. **(B)** Quantification of the histogram plots showing the median fluorescent intensity (MFI) normalized to MFI (set as 1) of the control. Before normalization, the MFI values of the negative controls were subtracted. **(C)** Viability of THP-1 cells was measured by the intensity of eFluor780 viability dye staining after one, two or three weeks of different concentrations of tunicamycin treatment (n=1).
